# Bat target tracking strategies for prey interception

**DOI:** 10.1080/19420889.2021.1898751

**Published:** 2021-03-12

**Authors:** Angeles Salles, Clarice A. Diebold, Cynthia F. Moss

**Affiliations:** Psychological and Brain Sciences, Johns Hopkins University, Baltimore, MD, USA

**Keywords:** Echolocation, predictive tracking, steering laws, echolocation, parallel navigation

## Abstract

Insectivorous bats capture their prey in flight with impressive success. They rely on the echoes of their own ultrasonic vocalization that yield acoustic snapshots, which enable target tracking on a rapid time scale. This task requires the use of intermittent information to navigate a dynamically changing environment. Bats may solve this challenging task by building internal models that estimate target velocity to anticipate the future location of a prey item. This has been recently tested empirically in perched bats tracking a target moving across their acoustic field. In this report, we build on past work to propose a new model that describes bat flight trajectories employing predictive strategies. Furthermore, we compare this model with a previous model of bat target interception that has also been employed by some visually guided animals: parallel navigation.

**Abbreviations:** HTTP, Hybrid Target Trajectory Prediction; CATD, Constant Absolute Target Direction; CB, Constant Bearing; PN, Parallel Navigation

## Introduction

Insectivorous bats are highly effective aerial predators that use echolocation to track and capture small insect prey in flight. Echolocation is an active sensing system by which bats probe their surroundings with ultrasonic vocalizations and analyze the features of the returning echoes that reflect from objects in the environment [1–3]. Bats are engaged in an evolutionary arms race with insect prey that has led to their use of sophisticated tracking strategies to counter evasion. Unlike other predators that use vision as their primary sensory system, and thus have a continuous stream of stimulus information to track moving targets, bats must compute the 3D trajectories of flying insects from sequences of echo snapshots [4]. Acoustic and neural delays that accumulate between the time the bat emits a call and executes a motor response could compromise successful execution of this task [5]. If the bat relied exclusively on location information from the last returning echo to orient to a target, the computed position of the prey item would be obsolete by the time the bat initiated appropriate motor responses for capture. In a recent study, we trained big brown bats, *Eptesicus fuscus*, to perch on a platform and track a target that moved laterally across its sonar field [6]. We quantified the direction of the bat’s head aim and sonar call rate and developed mathematical models showing that big brown bats can anticipate a target’s future position. These results support the conclusion that bats build internal models of target motion, challenging an earlier proposal from our lab that bats can use a strategy, that offsets acoustic and neural delays by maintaining a constant absolute target direction (CATD) to plan prey interception without invoking an internal model of target motion [7]. This strategy could be implemented by nulling changes in head direction or the apparent motion of acoustic background, which would not necessarily require an internal model of target motion; however, these alternatives were not directly assessed. Interestingly, the CATD reported in bats shows similarities with parallel navigation (PN) employed by guided missiles [8], and visually guided animals like hawks [9] and robber flies [10]. Parallel navigation relies on keeping each line-of-sight vector parallel to one another during the pursuit, and it enables interception as long as the predator is moving faster than the prey and the line-of-sight vector is shortened over time [8,11]. If both prey and predator are moving in straight lines, PN can be achieved by maintaining a fixed angle between the line-of-sight to the prey and the movement vector of the predator, and in this case PN is equivalent to constant bearing (CB) [10,11]. If the trajectory lines are not straight, PN occurs naturally if a time-optimal CATD strategy is used, in which the bearing changes depending on the target velocity and the predator-prey relative position. Past demonstration of CATD in bats does not incorporate the discrete and dynamic sampling of target location, essential components of echolocation [7,9]. Dynamic sonar sampling is evidenced by the bat’s active adjustments of echolocation call rate as it transitions through the search, approach and attack phases of insect pursuit, and is featured in the steering law described in [12]. This law reveals an adjustable linkage between the bat’s sonar beam axis and its flight path (acoustic gaze angle), and its flight turn rate; modulated by a gain factor that varies with the stage of insect pursuit, as defined by echolocation call rate [12]. As shown in [6], a predictive model of target motion that assumes the bat estimates target velocity through a sequence of echo snapshots and further adjusts its head angle by a fixed offset can account for the sonar tracking behavior of a perched animal. Here, we extend this model from a perched bat to explore its validity in accounting for target tracking and interception of a flying bat, by combining target velocity estimates from echo snapshots and a fixed head angle offset with the adaptive steering law [6,12]. This new model, referred to here as a hybrid target trajectory prediction (HTTP) strategy, makes use of an internal model of target motion in aerial predator-prey encounters. We compare HTTP with a variant of CATD that also takes into account the discrete nature of the acoustic information that the bats rely on to execute target tracking in flight.

## Results

**Figure 1.**
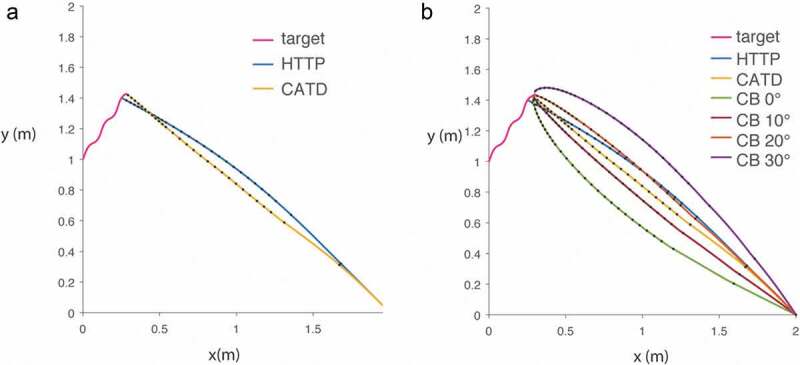
Model trajectories of a bat in pursuit of a prey item (pink line). Black dots represent echolocation signals being emitted at changing rates as the bat approaches the target. (a) Path corresponding to a predictive model that anticipates target position using acoustic snapshots (HTTP, blue line) and constant absolute target direction model (CATD variant, yellow line). (b) Comparison of HTTP and CATD with CB with different bearing angles ranging from 0 to 30 degrees

We combined two empirically tested models [6,12] to generate a new hybrid model that can describe the flight path of a free-flying bat that is actively echolocating in pursuit of a target. We call this combined model Hybrid Target Trajectory Prediction (HTTP). We compute the bat’s sonar beam axis (head aim) at a moving target by estimating target velocity from five successive echoes and a fixed head angle offset 6.2 degrees (these parameters reliably reproduce the predictive tracking behavior of perched bats) [6]. The body of the bat follows the bat’s head aim, as described in [12], with a gain ‘k’ and delay ‘tau’ that depends on the distance of the bat to the prey (k = 3.21, tau = 148 ms during the search phase; k = 4.24, tau = 128 ms during the approach phase; k = 6.26, tau = 96 ms during the attack phase; phase transition distances as described in [13]). Collectively, these parameters determine the model bat’s flight path. For the purpose of this work, we mimicked the echolocation behavior of *Eptesicus fuscus*, a bat species that decreases pulse interval (PI) as it approaches a target, through three phases: Search (PI = 107.5 ms), Approach (PI = 13.3 ms), and Attack (PI = 6.5 ms) [12]. We assumed the bat’s velocity was 4.2 m/s, representing its average velocity, as reported in [14]. The target was modeled as a coleopteran, which has been described as the preferred food source of this bat species [15], flying at an average of 1 m/s, as described in previous reports [16,17]. The flight path for both bat and prey is computed in 1 ms increments. We also computed the flight path of a bat employing CATD, but instead of continuous information about the target location, the bat receives intermittent measurements, driven by discrete echolocation sampling, that yield target velocity estimates from the last five echo returns. Thus, both the CATD variant shown here and HTTP take into account the discrete sampling of target position through echolocation. Figure 1(a) shows an example trajectory for a prey item (dashed line), the flight path as calculated by our variant of CATD (yellow line) and the flight path based on the HTTP model (blue line). We compared these trajectories to CB, setting the constant bearing angle to a range from 0 to 30 degrees, in 10º increments (Figure 1(b)).

## Conclusion

Previous research [7] showed that the bat’s prey capture behavior can be described by a flight trajectory that minimizes time to capture, given the instantaneous position and velocity vector of the prey item (CATD). This strategy showed a better fit with the bat’s flight path selection than CB. However, CATD fails to take into account the discrete nature of echolocation that yields intermittent information about the prey’s location. This may explain why CATD produces a better fit to the bat’s flight behavior in the last phase of insect pursuit when echolocation sample rate increases. We recently showed that an anticipatory model predicts the head aim (aligned with the sonar beam axis) of a perched bat [6]. This model incorporated naturalistic sampling of sonar target location through echolocation, and we extended it to build a Hybrid Target Trajectory Prediction (HTTP) model by applying a steering law [8] to predict performance in aerial prey capture [12]. We also extended CATD to incorporate discrete updates on target position through echolocation and compared the predicted flight path with that of HTTP and CB trajectories. We observed that different models generated similar trajectories to intercept prey, yet they are sufficiently distinct that future research is needed to determine which model produces an overall better fit to observed flight behavior in free-flying bats, or whether different capture phases are most accurately described by different models. Furthermore, it is plausible that bats may also integrate estimates of target mass and flight parameters to contend with erratic prey maneuvers. We aim to further compare these models in the lab by training bats to capture moving prey in flight and compare the reconstructed trajectories to those proposed by the models presented in this report. Our future directions will include the expansion of target tracking models and the use of neural networks to explore the extent to which an animal’s behavioral state (e.g. search, approach or attack phases) and a target’s evasive maneuvers (e.g. loops or dives) drive switches in tracking and interception strategies. These advances will further the understanding of predator-prey interactions, auditory motion tracking in mammals and inspire algorithms for automated tracking of dynamic auditory objects.

## References

[1] Simmons JA. The resolution of target range by echolocating bats. J Acoust Soc Am. 1973;54:157–173. 4738624 10.1121/1.1913559

[2] Simmons JA, Kick SA, Lawrence BD, et al. Acuity of horizontal angle discrimination by the echolocating bat, Eptesicus fuscus. J Comp Physiol A. 1983;153:321–330.

[3] Moss CF, Surlykke A. Auditory scene analysis by echolocation in bats. J Acoust Soc Am. 2001;110:2207–2226. 11681397 10.1121/1.1398051

[4] Diebold CA, Salles A, Moss CF. Adaptive echolocation and flight behaviors in bats can inspire technology innovations for sonar tracking and interception. Sensors. 2020;20:2958. 10.3390/s20102958 PMC7285367 32456142

[5] Moss CF, Chiu C, Surlykke A. Adaptive vocal behavior drives perception by echolocation in bats. Curr Opin Neurobiol. 2011;21:645–652. 21705213 10.1016/j.conb.2011.05.028 PMC3178000

[6] Salles A, Diebold CA, Moss CF. Echolocating bats accumulate information from acoustic snapshots to predict auditory object motion. Proc Natl Acad Sci U S A. 2020;117:29229–29238. 33139550 10.1073/pnas.2011719117 PMC7682551

[7] Ghose K, Horiuchi TK, Krishnaprasad PS, et al. Echolocating bats use a nearly time-optimal strategy to intercept prey. PLoS Biol. 2006;4:e108. 16605303 10.1371/journal.pbio.0040108 PMC1436025

[8] Shneydor NA. Missile guidance and pursuit: kinematics, dynamics and control. Cambridge, United Kingdom: Elsevier; 1998.

[9] Kane SA, Fulton AH, Rosenthal LJ. When hawks attack: animal-borne video studies of goshawk pursuit and prey-evasion strategies. J Exp Biol. 2015;218:212–222. 25609783 10.1242/jeb.108597 PMC4302165

[10] Wardill TJ, Fabian ST, Pettigrew AC, et al. Strategy in a miniature robber fly with extreme visual acuity. Curr Biol. 2017;27:854–859. 28286000 10.1016/j.cub.2017.01.050 PMC5364399

[11] Fabian ST, Sumner ME, Wardill TJ, et al. Interception by two predatory fly species is explained by a proportional navigation feedback controller. J R Soc Interface. 2018;15(147):20180466. 10.1098/rsif.2018.0466 PMC6228472 30333249

[12] Ghose K, Moss CF. Steering by hearing: a bat’s acoustic gaze is linked to its flight motor output by a delayed, adaptive linear law. J Neurosci. 2006;26:1704–1710. 16467518 10.1523/JNEUROSCI.4315-05.2006 PMC3437256

[13] Kick SA, Simmons JA. Automatic gain control in the bat’s sonar receiver and the neuroethology of echolocation. J Neurosci. 1984;4:2725–2737. 6502201 10.1523/JNEUROSCI.04-11-02725.1984 PMC6564721

[14] Sandig S, Schnitzler H-U DA. Echolocation behaviour of the big brown bat (Eptesicus fuscus) in an obstacle avoidance task of increasing difficulty. J Exp Biol. 2014;217:2876–2884. 24902745 10.1242/jeb.099614

[15] Feldhamer GA, Carter TC, Whitaker JO. Prey consumed by eight species of insectivorous bats from Southern illinois. Am Midl Nat. 2009;162:43–51.

[16] Farisenkov SE, Lapina NA, Petrov PN, et al. Extraordinary flight performance of the smallest beetles. Proc Natl Acad Sci USA. 2020;117:24643–24645. 32958659 10.1073/pnas.2012404117 PMC7547253

[17] Zhang H, Teng X, Luo Q, et al. Flight and walking performance of dark black chafer beetle holotrichia parallela (Coleoptera: scarabaeidae) in the presence of known hosts and attractive nonhost plants. J Insect Sci. 2019;19. DOI:10.1093/jisesa/iez019. PMC6415723 30865781

